# Supplementary material for: Post-transcriptional regulation across human tissues
Source: PLoS Comput Biol. 2017 May 8;13(5):e1005535. doi: 10.1371/journal.pcbi.1005535 (PMC5440056; doi:10.1371/journal.pcbi.1005535)
Supplement: S1 Table — Pearson correlations between two estimates of the median rPTR ratios for all GO terms indicate reproducible effects in all tissues. As in Fig 2, rPTR estimates are derived using independent data sources. The lower and upper estimates are the endpoints of the 95% confidence interval. (PDF) [file pcbi.1005535.s001.pdf]

	adrenal	colon	esophagus	kidney	liver	lung	ovary	pancreas	prostate	testis
Corr.	0.38	0.33	0.14	0.34	0.16	0.14	0.07	0.18	0.39	0.27
Lower	0.34	0.30	0.09	0.31	0.12	0.10	0.03	0.13	0.36	0.24
Upper	0.41	0.36	0.19	0.38	0.20	0.18	0.10	0.22	0.42	0.31

Table S1. Estimates of relative protein-to-RNA (rPTR) ratio for GO terms reproduce across different datasets Pearson correlations between two estimates of the median rPTR ratios for all GO terms indicate reproducible effects in all tissues. As in Fig.2, rPTR estimates are derived using independent data sources. The lower and upper estimates are the endpoints of the 95% confidence interval.
