# Supplementary material for: Post-transcriptional regulation across human tissues
Source: PLoS Comput Biol. 2017 May 8;13(5):e1005535. doi: 10.1371/journal.pcbi.1005535 (PMC5440056; doi:10.1371/journal.pcbi.1005535)
Supplement: S2 Table — Correlations between the two estimates of rPTR ratios for all genes indicate reproducible effects in all tissues. The rPTR ratios were estimated independently from different datasets (as in Fig 2). The lower and upper estimates are the endpoints of the 95% confidence interval. (PDF) [file pcbi.1005535.s002.pdf]

	adrenal	colon	esophagus	kidney	liver	lung	ovary	pancreas	prostate	testis
Corr.	0.39	0.36	0.18	0.32	0.49	0.18	0.07	0.22	0.38	0.27
Lower	0.36	0.33	0.13	0.28	0.46	0.14	0.03	0.18	0.35	0.24
Upper	0.42	0.39	0.22	0.35	0.52	0.22	0.10	0.27	0.42	0.31

Table S2. Estimates of relative protein-to-RNA (rPTR) ratio for genes reproduce across different datasets Correlations between the two estimates of rPTR ratios for all genes indicate reproducible effects in all tissues. The rPTR ratios were estimated independently from different datasets (as in Fig 2). The lower and upper estimates are the endpoints of the 95% confidence interval.
