# Supplementary material for: Post-transcriptional regulation across human tissues
Source: PLoS Comput Biol. 2017 May 8;13(5):e1005535. doi: 10.1371/journal.pcbi.1005535 (PMC5440056; doi:10.1371/journal.pcbi.1005535)
Supplement: S1 Fig — (a-c, top row), protein versus mRNA in kidney, liver and prostate. (d-f, middle row) protein versus scaled mRNA in kidney, liver and prostate. The only difference from the top row is that the mRNA was scaled by the median PTR. (g-i, bottom row) protein fold changes versus the corresponding mRNA fold changes between the tissues indicated on the top. While scaled mRNA is predictive of the absolute protein levels the accuracy of these predictions does not generally reflect the accuracy of protein fold-changes across tissues that are predicted from the corresponding mRNA fold-changes. RNA fold changes in (g-i, bottom row) were computed between the mRNA levels without PTR scaling. (PDF) [file pcbi.1005535.s005.pdf]

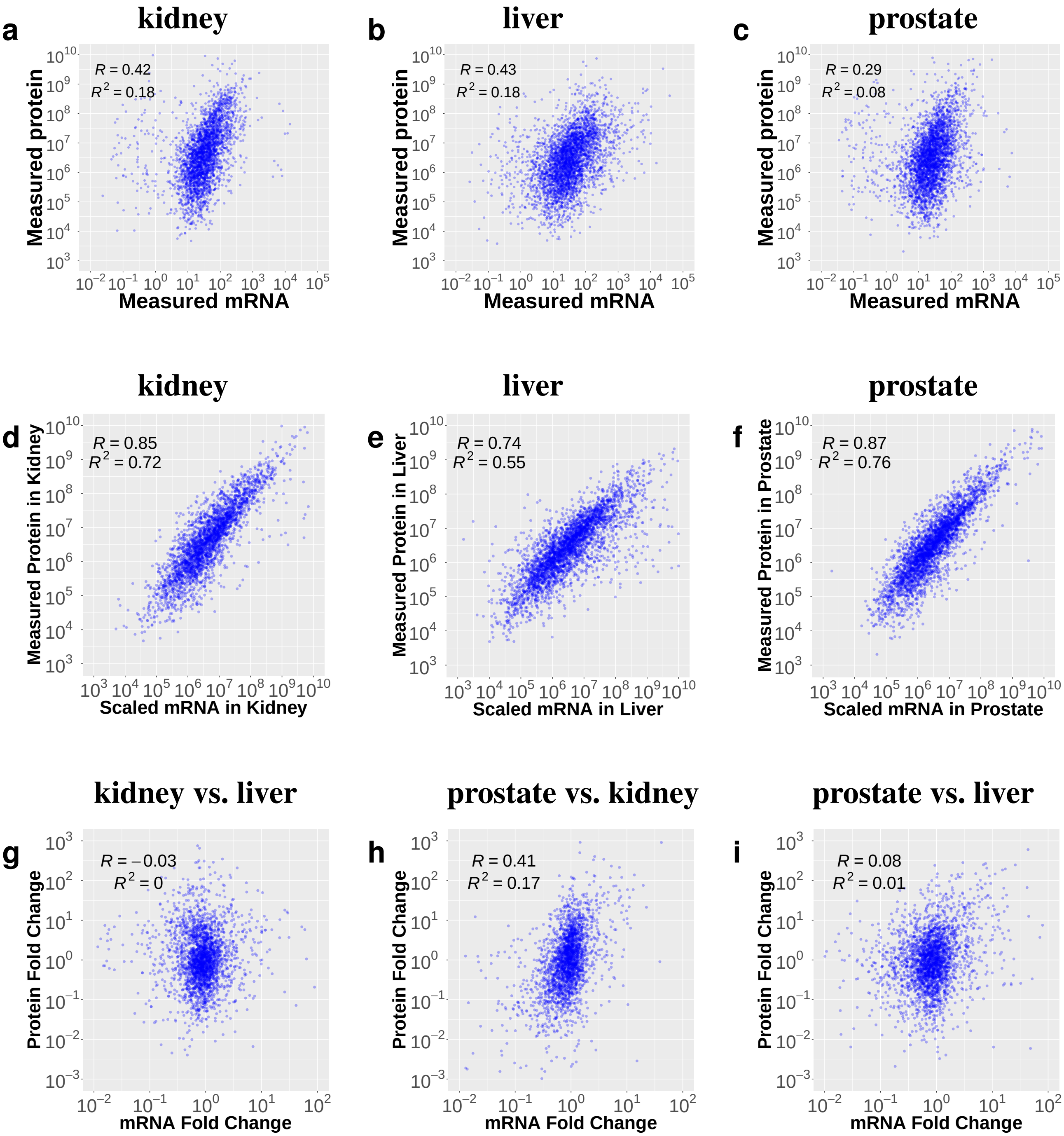

Figure S1. The total protein variance explained by scaled mRNA levels is not indicative of the correlations between mRNA and protein fold-changes across the corresponding tissue pairs. (a-c, top row), protein versus mRNA in kidney, liver and prostate. (d-f, middle row) protein versus scaled mRNA in kidney, liver and prostate. The only difference from the top row is that the mRNA was scaled by the median PTR. (g-i, bottom row) protein fold changes versus the corresponding mRNA fold changes between the tissues indicated on the top. While scaled mRNA is predictive of the absolute protein levels the accuracy of these predictions does not generally reflect the accuracy of protein fold-changes across tissues that are predicted from the corresponding mRNA fold-changes. RNA fold changes in (g-i, bottom row) were computed between the mRNA levels without PTR scaling.
