# Supplementary material for: Post-transcriptional regulation across human tissues
Source: PLoS Comput Biol. 2017 May 8;13(5):e1005535. doi: 10.1371/journal.pcbi.1005535 (PMC5440056; doi:10.1371/journal.pcbi.1005535)
Supplement: S2 Fig — (a) The distributions of across-tissues correlations for gene sets defined by the gene ontology are shown as boxplots. The reliability of RNA and protein are estimated as the correlations between estimates from different datasets. (b) For each gene set, the median RNA-protein correlation was corrected by the median reliabilities and the results shown as a boxplot. Differences between RNA-protein correlations for different gene-sets cannot be explained simply by differences in the reliabilities. (PDF) [file pcbi.1005535.s006.pdf]

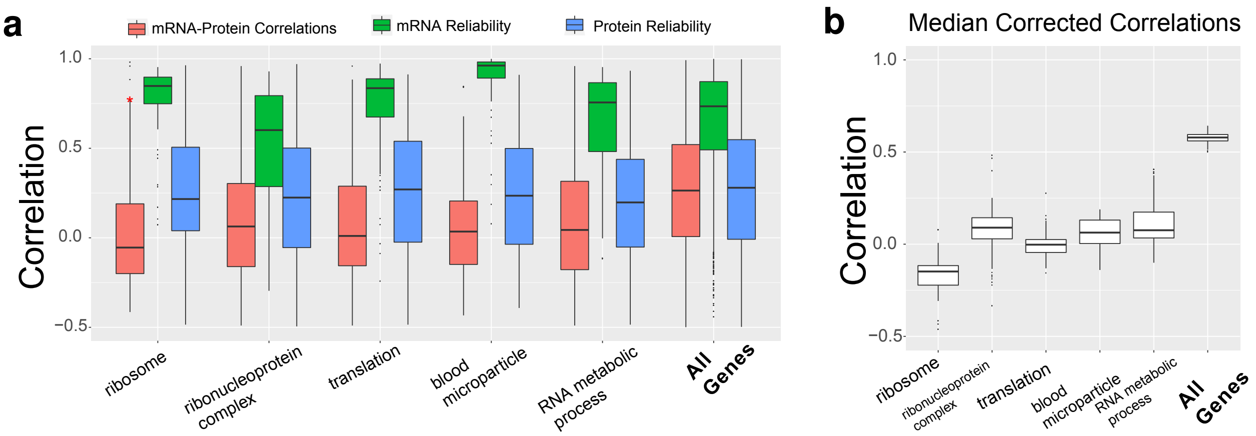

Figure S2. Fraction of across-tissues variability in protein levels explained by RNA variability for different functional gene sets. (**a**) The distributions of across-tissues correlations for gene sets defined by the gene ontology are shown as boxplots. The reliability of RNA and protein are estimated as the correlations between estimates from different datasets. (**b**) For each gene set, the median RNA-protein correlation was corrected by the median reliabilities for different subsets and the results shown as a boxplot. Differences between RNA-protein correlations for different sets cannot explained simply by differences in the reliabilities.
