# Supplementary material for: Post-transcriptional regulation across human tissues
Source: PLoS Comput Biol. 2017 May 8;13(5):e1005535. doi: 10.1371/journal.pcbi.1005535 (PMC5440056; doi:10.1371/journal.pcbi.1005535)
Supplement: S3 Fig — The x-axes shows estimates from Wilhelm et al. [20] and the y-axes estimates from Kim et al. [21]. (PDF) [file pcbi.1005535.s007.pdf]

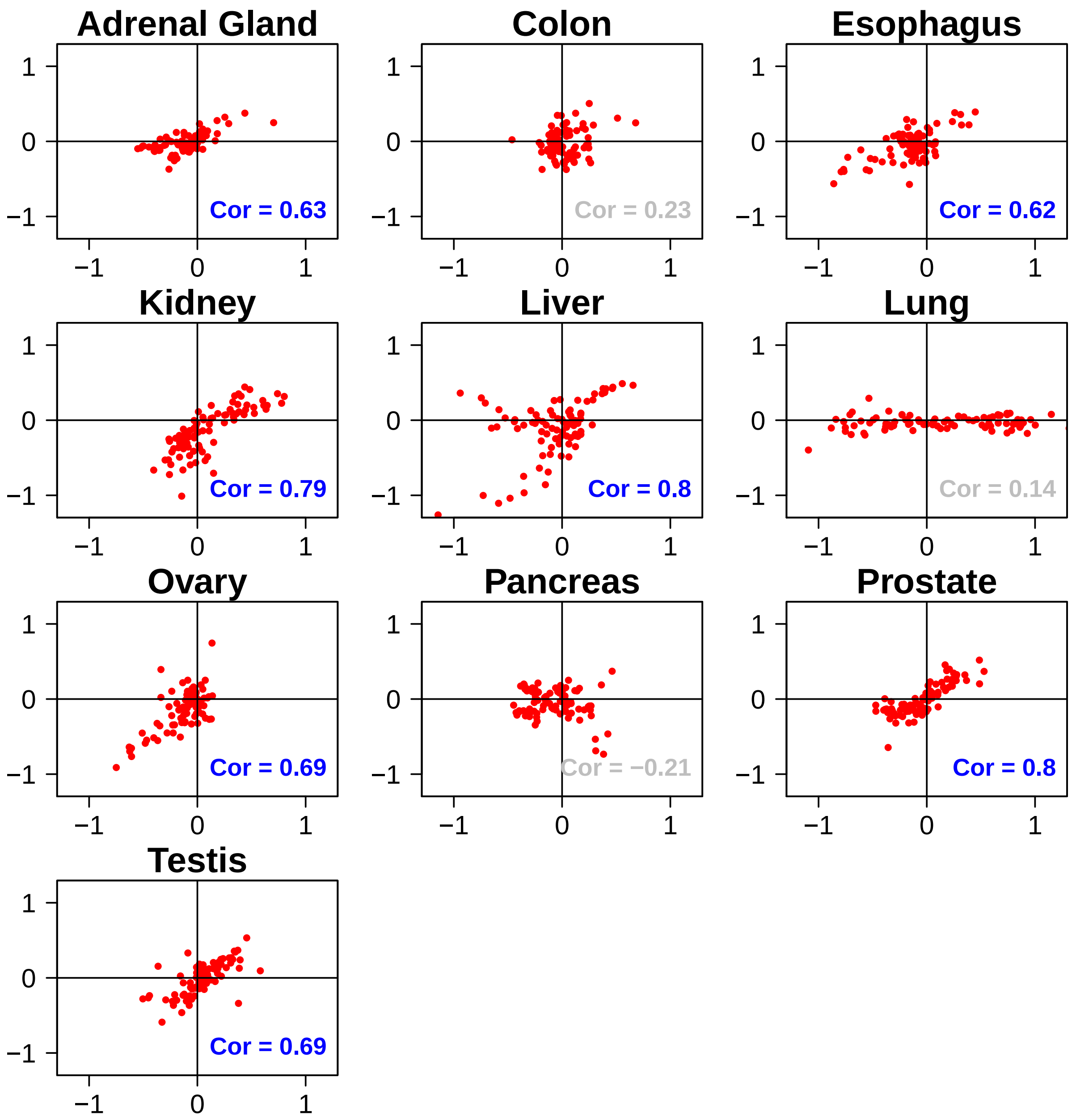

Figure S3. Reproducibility of rPTR ratios estimated from different datasets The x-axes shows estimates from Wilhelm et al. [20] and the y-axes estimates from Kim et al. [21].
